# Leaf Dynamics of *Panicum maximum *under Future Climatic Changes

**DOI:** 10.1371/journal.pone.0149620

**Published:** 2016-02-19

**Authors:** Carlos Henrique Britto de Assis Prado, Lívia Haik Guedes de Camargo-Bortolin, Érique Castro, Carlos Alberto Martinez

**Affiliations:** 1 Department of Botany, Federal University of São Carlos, São Carlos, São Paulo, Brazil, 13565–905; 2 Department of Biology, FFCLRP, University of São Paulo, Ribeirão Preto, São Paulo, Brazil, 14040–901; National University of Ireland - Galway, IRELAND

## Abstract

*Panicum maximum* Jacq. ‘Mombaça’ (C4) was grown in field conditions with sufficient water and nutrients to examine the effects of warming and elevated CO_2_ concentrations during the winter. Plants were exposed to either the ambient temperature and regular atmospheric CO_2_ (Control); elevated CO_2_ (600 ppm, eC); canopy warming (+2°C above regular canopy temperature, eT); or elevated CO_2_ and canopy warming (eC+eT). The temperatures and CO_2_ in the field were controlled by temperature free-air controlled enhancement (T-FACE) and mini free-air CO_2_ enrichment (miniFACE) facilities. The most green, expanding, and expanded leaves and the highest leaf appearance rate (LAR, leaves day^-1^) and leaf elongation rate (LER, cm day^-1^) were observed under eT. Leaf area and leaf biomass were higher in the eT and eC+eT treatments. The higher LER and LAR without significant differences in the number of senescent leaves could explain why tillers had higher foliage area and leaf biomass in the eT treatment. The eC treatment had the lowest LER and the fewest expanded and green leaves, similar to Control. The inhibitory effect of eC on foliage development in winter was indicated by the fewer green, expanded, and expanding leaves under eC+eT than eT. The stimulatory and inhibitory effects of the eT and eC treatments, respectively, on foliage raised and lowered, respectively, the foliar nitrogen concentration. The inhibition of foliage by eC was confirmed by the eC treatment having the lowest leaf/stem biomass ratio and by the change in leaf biomass-area relationships from linear or exponential growth to rectangular hyperbolic growth under eC. Besides, eC+eT had a synergist effect, speeding up leaf maturation. Therefore, with sufficient water and nutrients in winter, the inhibitory effect of elevated CO_2_ on foliage could be partially offset by elevated temperatures and relatively high *P*. *maximum* foliage production could be achieved under future climatic change.

## Introduction

Currently, the atmospheric CO_2_ concentration reaches above 403 ppm, a significant increasing since 1960, when 320 ppm was recorded at the Mauna Loa Observatory in Hawaii [1]. This is highly important because CO_2_ is a greenhouse gas and causes global warming and climate change. Accordingly, the average surface temperature of the Earth has consistently increased since 1980 [2]. The Intergovernmental Panel on Climate Change [3] uses a range of future scenarios based on social, technological, economic, and environmental conditions to predict the atmospheric CO_2_ concentration and the Earth’s surface temperature. The IPCC projects an increase in the surface temperature of 2°C by 2050 with very heterogeneous stages of human development in a scenario named A2. In this scenario, the concentration of atmospheric CO_2_ will be around 600 ppm [4]. Heating and the greater availability of CO_2_ will affect plant growth, development, and yields [5], [6]. Since forages are directly exposed to atmosphere conditions in the field, climate change will significantly impact the management of pastures [7].

Brazil has been the world’s largest meat exporter since 2008, with 200 million head of cattle, and the value of the meat and milk production chains has been estimated at US$31 billion [8]. The favorable climate and large size of Brazil contribute to this success. However, despite the great importance of ranching, the effects of climate change on forages and their acclimation to the combined effects of elevated CO_2_ concentration and warming have not yet been evaluated under field conditions in Brazil. In fact, there is a multi-biome gap in experimental data from the tropics and subtropics on plant responses to future climatic changes [9], [10].

Plants clearly respond to increased CO_2_ concentrations, since even for some C4 species, the current concentration of atmospheric CO_2_ is below the CO_2_ saturation point for net photosynthesis [11]. Indeed, some results from open-top chamber studies have found that an elevated CO_2_ concentration can stimulate biomass production in C4 species [12], [13]. However, the net photosynthesis and growth of C4 species are less responsive than those of C3 species under elevated CO_2_ [14]. In contrast to physiological responses, information on morphological alterations in the leaf blades of C4 grasses under future climatic change is scarce. This is an important gap in knowledge because the anatomical and morphological traits of the leaf blades of *Panicum maximum* can affect animal preferences [15].

The purpose of this work was to determine the effects of future climate change on foliar growth and the stages of leaf development in tillers of Guinea grass (*Panicum maximum* Jacq.) following the A2 scenario. The major C4 grasses used as forage for cattle in Brazil belong to the genera *Panicum* and *Brachiaria* [15]. Our results will support pasture management in farms in future climate changes since the experiment was carried out under field conditions using a mini Free-air Carbon dioxide Enrichment (miniFACE) system and a Temperature Free-air Controlled Enhancement (T-FACE) system.

We hypothesized that a temperature increase of 2°C will stimulate foliage production in *P*. *maximum*, especially if there is no water shortage. This would be possible, especially in winter, because *P*. *maximum* is a C4 tropical forage with a high optimal leaf temperature for photosynthesis (30°C–40°C) [16], [17]. Heating probably stimulates the rate of leaf expansion, as found in C4 buffel grass *Cenchrus ciliaris*, resulting in higher foliar area and biomass [13]. On the other hand, an elevated CO_2_ concentration will promote little or no change in leaf area and leaf biomass of *P*. *maximum* since the C4 photosynthetic pathway already acts as a CO_2_ concentration mechanism [18]. Nonetheless, if leaf growth rate, leaf area, and leaf biomass will increase under warming in winter, the effect of elevated CO_2_ on heated leaves of *P*. *maximum* in the field would be hard to predict. Therefore, we tested the leaf dynamics of *P*. *maximum* under current conditions and the predicted A2 atmospheric scenario for the year 2050 by controlling the temperature and CO_2_ concentration in the field.

## Materials and Methods

### Experimental area, plant material, soil preparation, and sowing

The study was conducted on a 2500 m^2^ field at the campus of the University of São Paulo (USP) in Ribeirão Preto municipality, state of São Paulo, Brazil (21°10′S and 47°48′W, 800 m altitude). According to the Köppen-Geiger (KG), Ribeirão Preto shows the Aw classification, a tropical climate with rainy summer and dry winter [19], [20]. The KG classification is efficient in macroscale, so we also considered the Thornthwaite classification (TH) which is efficient in mesoscale [21]. According to TH, Ribeirão Preto city shows the B_2_rB’_4_a’ classification [22], a humid climate, with little or no water deficiency in dry season from April to September [22], [23]. The Camargo climate classification (CA) combines the simplicity of KG with the accuracy of TH using an agroclimatic zoning [21]. According to CA, Ribeirão Preto city shows the TR-SBi classification, a subhumid tropical climate with dry winter [21]. Historical data from 1982 to 2012 present the average annual temperature and rainfall of 21.9°C and 1508 mm, respectively [24]. For the period August-September, the historical data (1982–2012) show an average temperature of 21.2°C, with minimum and maximum temperatures of 14.2°C and 28.2°C, respectively [24].

According to the U.S. Department of Agriculture and "Embrapa Solos" in Brazil, the soil in the area is a dystrophic Red Latosol (Oxisol) [25]. The area is fenced, and soil analysis, contouring, railing, and soil pH correction by liming had been performed previously. The value of the average initial pH was 4.0 to 4.5 and remained at 5.0 to 5.5 after liming. Chemical soil fertilization happened after pH correction according to the initial nutrient availability. Therefore, the soil was appropriate and homogeneous at the time of planting.

Seeds of Guinea grass, *Panicum maximum* Jacq. (Poaceae), were planted into holes 30 cm apart in 12 m × 12 m plots. NPK 4-14-8 fertilizer was used at a dose of 1 t ha^-1^ applied into the holes during planting. Only three plants per hole were maintained after germination. Sixty days after planting, the tussocks were cut at a height of 30 cm from the ground. This trimming established a full, similar canopy among treatments as the standard practice for managing *P*. *maximum* in natural conditions.

### Trop-T-FACE system, watering, and treatments

The Trop-T-FACE system is a new combined Free-air Temperature and CO_2_ Controlled Enhancement system to evaluate the performance of tropical pastures under future climate change scenarios. The FACE component for elevated CO_2_ treatments under field conditions was a modified POPFACE sonic injection system of pure CO_2_ designed by our colleagues [26]. Sixteen plots were created inside rings with a diameter of 2 m. Eight of these rings were utilized for the treatment of elevated CO_2_ in the small FACE system named miniFACE. The eight rings of miniFACE were placed randomly within the experimental area so that no ring was less than 10 m from another to minimize CO_2_ cross-contamination.

CO_2_ was sampled at the center of each miniFACE ring by an air pump and monitored by a high-speed CARBOCAP infrared gas analyzer (IRGA) Model GMP 343 (Vaisala, Finland). Each IRGA monitored the CO_2_ concentration for manipulating the supply of pure CO_2_ gas in each ring. The amount of pure CO_2_ was controlled by varying the pressure within the gas release pipes with an automatic pressure regulator (SMC Corporation, ITV series, Japan). The flow of each valve was controlled by adjusting the valve tension, which was determined by a programmable control system using the microprocessor-based Proportional Integration Device (PID algorithm).

The PID algorithm integrated the CO_2_ concentration measured at the center of each miniFACE ring with the wind speed determined by an anemometer located 2 m above the ground in the center of the planted area. Then, the PID determined the voltage of the valve and thus the rate of release of pure CO_2_ in each ring. CO_2_ fumigation began at sunrise and ended at sunset each day from August 22 to September 20, 2013. All variables (CO_2_ concentration, valve voltage, and wind speed) were recorded every 5 seconds on a computer located in the central control of the miniFACE system inside a container near the field. Four separated rings had the regular environmental CO_2_ concentration and eight miniFACE rings had the elevated CO_2_ concentration of 600 ppm.

Four of the eight miniFACE rings also had an elevated canopy temperature treatment. The temperature free-air controlled enhancement component of the Trop-T-FACE changes the canopy temperature with infrared ceramic heaters controlled by a PID algorithm [27]. The heaters were model FTE-750-240 Salamander ceramic infrared heating elements, mounted on Salamander ALEX-F reflectors (Mor Electric, MI, USA). The heaters were suspended from a triangular aluminum pole system. There were six heaters per plot, with a heater at each point of a hexagon. To produce a consistent amount of shade in the warmed and reference plots, similar arrays of dummy heaters consisting of aluminum reflectors without a heating element were installed in the reference plots.

In the eight warmed plots, the heaters were maintained at 0.8 m above the canopy. The temperature of the canopy was measured using an infrared thermometer model SI-1H1-L20 (Apogee Instruments, USA). We used a PID algorithm installed in a CR1000 data logger with AM25T multiplexors (Campbell Scientific, USA) to control the voltage of the heaters [27]. To monitor and collect the data, we used LoggerNet software (Campbell Scientific, USA) installed on a computer. For communication, an NL201 network link interface (Campbell, Scientific, USA) and a wired Ethernet network connection were used with the data logger. There were two thermal canopy regimes: the regular temperature in the Control plots and the temperature elevated by 2°C above the regular canopy temperature in the warmed plots.

Soil water content and the soil temperature in each ring were monitored with Theta Probe soil moisture (ML2x) and temperature (ST2) sensors connected to a DL2e data logger (Delta-T Devices, UK), respectively. An automatic microclimatic station (WS-HP1) monitored and continuously stored the climatic data (temperature, relative humidity, irradiance, and precipitation) using specific sensors. Once the plants were grown under field conditions in the winter (dry season), soil and air moisture were monitored continuously. Water sprinklers were used for irrigating the entire area of all 16 plots during the period of the experiment in order to maintain the soil moisture near field capacity.

In summary, the design of this experiment to evaluate foliar growth and stages of leaf development in tillers of *P*. *maximum* included four treatments: ambient conditions (Control), an atmospheric CO_2_ concentration elevated to 600 ppm (eC), a canopy temperature elevated 2°C above the ambient temperature (eT), and both an elevated CO_2_ concentration and canopy temperature (eC+eT). Each treatment had four replicates, totaling 16 experimental units.

### Foliage measurements and data analysis

Foliage growth and stages of leaf development were set in the context of the tiller, the basic unit of growth in grasses. Three randomly equidistant tussocks of *P*. *maximum* were selected in each plot. Five tillers were chosen per tussock, resulting in 15 tillers per plot for assessing foliage. Since the experiment was performed with four replicates per treatment, 60 tillers were measured per treatment. Data collection took place on August 22 and 29 and September 3, 9, 12, 19, and 20 in 2013.

Foliage traits recorded on each date included the number of expanded and expanding leaves (with and without visible ligule, respectively), the number of senescent leaves (with at least 50% of the area yellowing), and the length of the expanding leaves. From these measurements, average values per tiller were obtained in each treatment, and the following parameters were calculated.

The number of leaves that emerged in each period between data collection days was named the leaf appearance rate (LAR, day^-1^). We obtained LAR as the number of leaves from the current data collection day minus the number of leaves from the previous data collection day, with only positive values considered. The final LAR in a corresponding treatment was obtained by summing all positive values from each measurement time divided by the number of days in the entire experiment (30 days). This parameter indicated the rate of the appearance of leaves per tiller. Among the original 60 tillers marked per treatment, 58, 56, 56, and 57 in the Control, eC, eT, and eC+eT treatments, respectively, were usable for obtaining the average value of LAR.

The leaf elongation rate (LER, cm day^-1^) was calculated as the length of expanding leaves on the current data collection day minus the length of these leaves from the previous data collection day, divided by the number of days between the two data collection times. Among the original 60 marked tillers per treatment, 59, 59, 60, and 58 in the Control, eC, eT, and eC+eT treatments, respectively, were usable for obtaining the average value of LER through the end of the experiment. An average value was calculated for the entire experimental period to show how fast the expanding leaves grew in length per tiller per day.

The combined number of expanded and expanding leaves (leaves with and without a ligule, respectively) resulted in the number of green leaves per tiller (NGL), disregarding senescent leaves. Senescent leaves were identified as those with more than 50% of their area without green color. The NGL was calculated as the total number of expanded and expanding leaves on each data collection day. An average NGL value was obtained for the entire experimental period to show how many functional leaves were maintained in each treatment. The total number of leaves considered was 349, 351, 358, and 349 in the Control, eC, eT, and eC+eT treatments, respectively. From the seven data collection days, it was possible to calculate the average number of green, expanded, expanding, and senescent leaves per tiller in each treatment.

From the data collection days, it was possible to compute the expanded, expanding, and senescent leaves accumulated per tiller. The accumulated values were obtained as the number of leaves on the first data collection day plus the number of leaves on subsequent data collection days. Trimming at the beginning of the experiment cut some expanding and expanded leaves, causing a permanent, typical scar on the leaf blade. In contrast, leaves that expanded after pruning had a typical intact apex. Therefore, it was possible to count the number of intact and cut green leaf blades among the expanded and expanding leaves per tiller. The leaf area of *P*. *maximum* was measured using the WinDIAS Leaf Image Analyses System (Delta-T, UK). The marked tillers of *P*. *maximum* in the field were collected intact by cutting their stem base at the ground level at the end of the experiment (21 September). The leaves were detached, and the area per tiller was measured. The foliage of each identified tiller was dried separately in a stove with forced air circulation at 60°C to obtain the leaf biomass. In addition, the foliar nitrogen content, N%, was determined in each ring using expanded and expanding leaves (the green leaves) by the semi-micro-Kjeldahl methodology [28]. Overall, four measurements of foliar nitrogen were carried out, and the average N% was obtained for each treatment at the end of the experiment.

Statistical analyses were performed with the open software BioEstat, version 5.3 (Instituto Mamirauá, Brazil). A D'Agostino-Pearson test was used to verify whether data were normally distributed. Since the datasets did not show a normal distribution, the non-parametric Mann-Whitney test was used to comparing the datasets among treatments. Significance was determined at p < 0.1 for LAR, LER, and foliar nitrogen content and p < 0.05 for the others foliage measurements in order to compare the datasets among treatments. Non-linear trend lines created in Microsoft Excel^™^ were used to perform the correlation analysis between leaf area and leaf mass.

## Results

**Fig 1:**
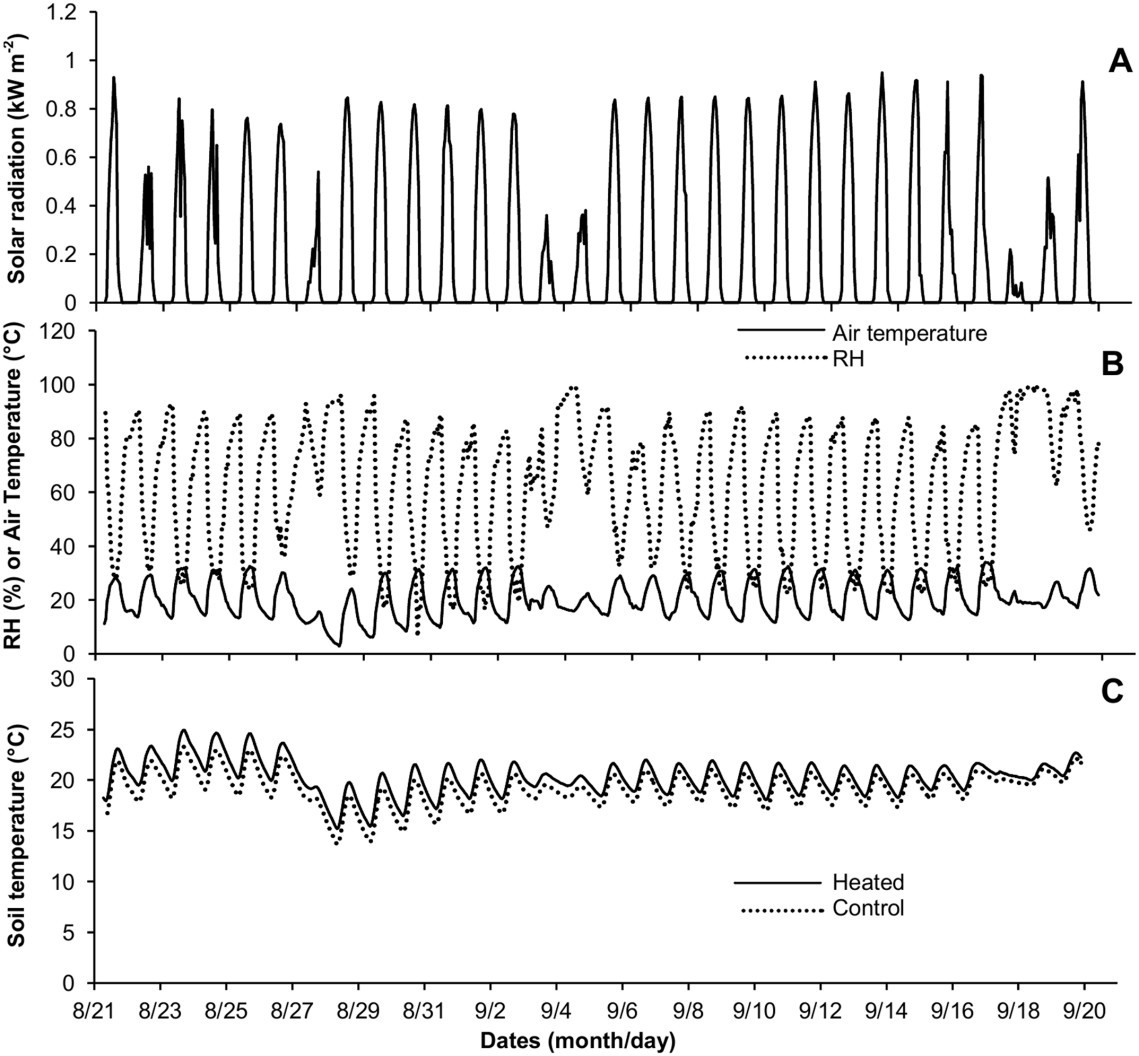
Daily courses of meteorological conditions and soil temperature during the period of the experiment, from August 22 to September 20, 2013. A—Total solar radiation. B—Air relative humidity (RH) and air temperature. C—Soil temperature in control and heated plots.

Days were usually cloud-free, with solar radiation reaching nearly 1 kW m^-2^ around midday during the experiment (Fig 1A). Nonetheless, cloudy days with solar radiation of around 0.5 kW m^-2^ occurred during September 3–4 and September 18–19 in 2013. Thus, air temperature was lower and relative air humidity was higher than on typical days (Fig 1B). On August 28, 2013, an unusually cold air temperature of 3°C at predawn, when the soil temperature reached the lowest value (Fig 1B). The soil temperature varied between 15 and 25°C in and the eT and Control treatments (Fig 1C). Only during the early morning, the soil temperature was almost the same in the two treatments. However, after 10:00 A.M., the soil temperature in eT was approximately 2°C above the Control (Fig 1C).

**Fig 2:**
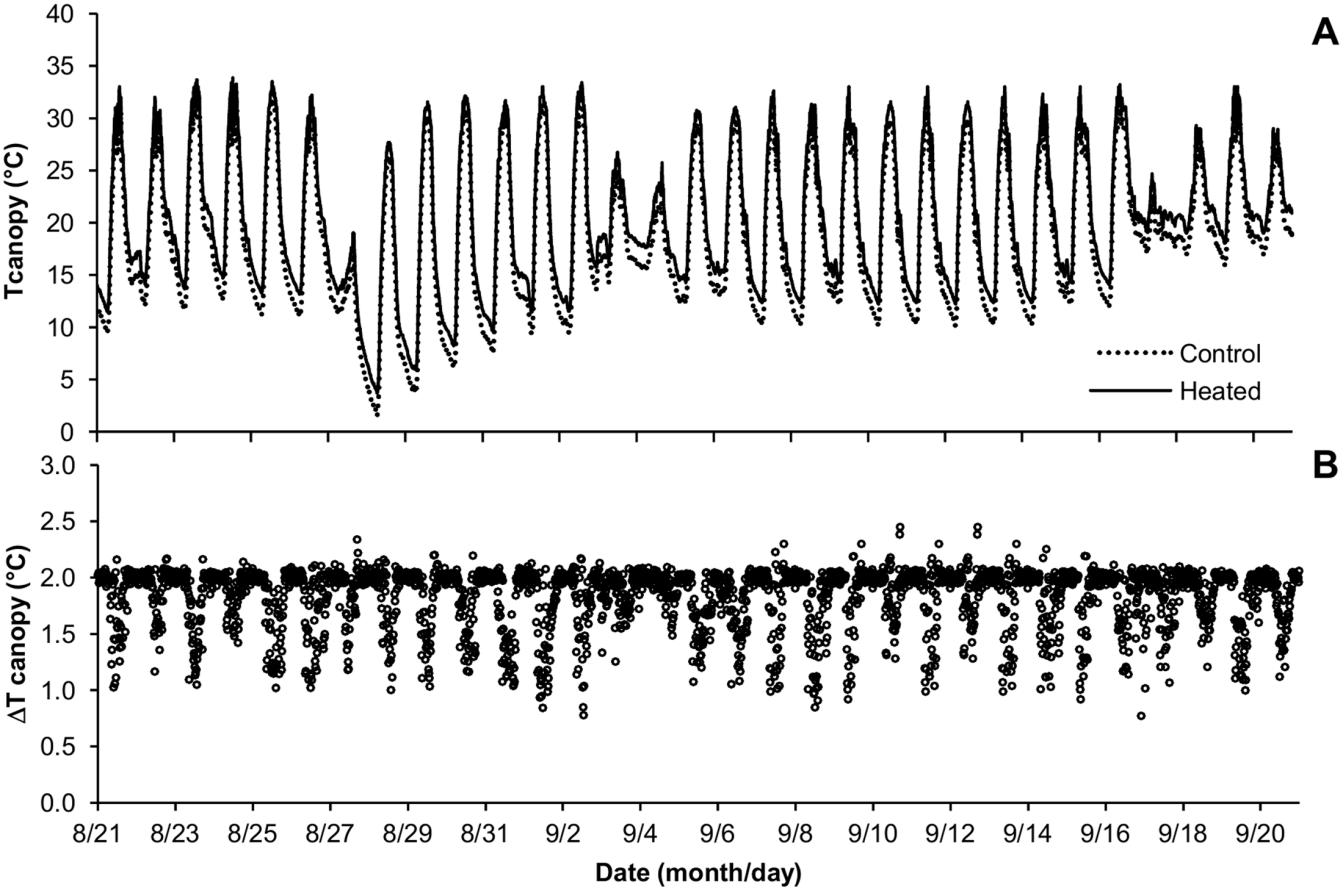
Canopy temperature during the period of the experiment, from August 22 to September 20, 2013. A—Canopy temperature of heated (solid line) and Control (dashed line) treatments. B—Difference (ΔT canopy) between heated and regular canopy temperatures indicating the deviations from the target elevated temperature of 2°C above the Control.

The canopy temperature in the Control and eT treatments was usually between 30°C and 10°C during the day (Fig 2A). Nonetheless, a colder period occurred on August 26–27, when the canopy temperature was as 3°C at night (Fig 2A). Despite the 20°C variation during a day, the target canopy temperature of +2°C in the eT treatment was achieved throughout the experiment, especially during the night (Fig 2B). The differences in canopy temperature between the Control and eT treatments reached values below the set point temperature (+2°C) for short periods because of leaf transpiration, particularly at midday (Fig 2B).

**Fig 3:**
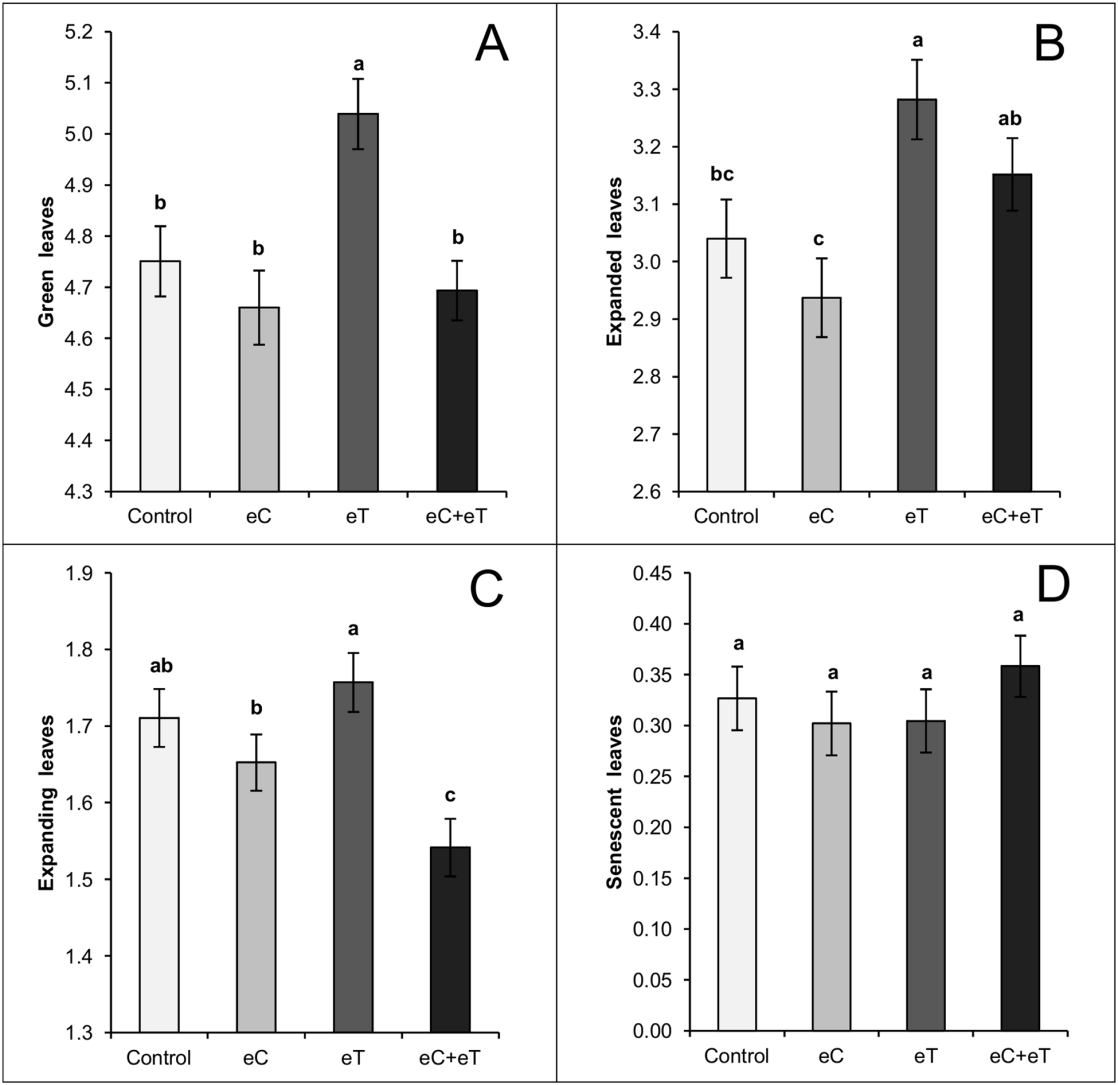
Number of green (A), expanded (B), expanding (C), and senescent (D) leaves per tiller of *Panicum maximum* under ambient CO_2_ and canopy temperature (Control), under an elevated CO_2_ concentration of 600 ppm (eC), under an elevated canopy temperature of +2°C (eT), and under both treatments (eC+eT). Bars indicate average values and lines at the top of bars indicate the standard error. Different letters above bars indicate significant differences among datasets according to a Mann-Whitney test at p < 0.05.

Compared to the Control, the eT treatment showed a significant (p < 0.05) increase in green (Fig 3A) and expanded leaves (Fig 3B). There was an unanticipated inhibitory effect on foliage in the eC+eT treatment, resulting in the significantly lowest number of expanding leaves (Fig 3C). Even with these significant differences in foliar dynamics, the number of senescent leaves was not affected by any treatment (Fig 3D). It is worth noting that, despite the non-significant difference at p < 0.05, the number of green, expanded, and expanding leaves were lower in the eC than in the Control treatment (Fig 3A, 3B and 3C, respectively).

**Fig 4:**
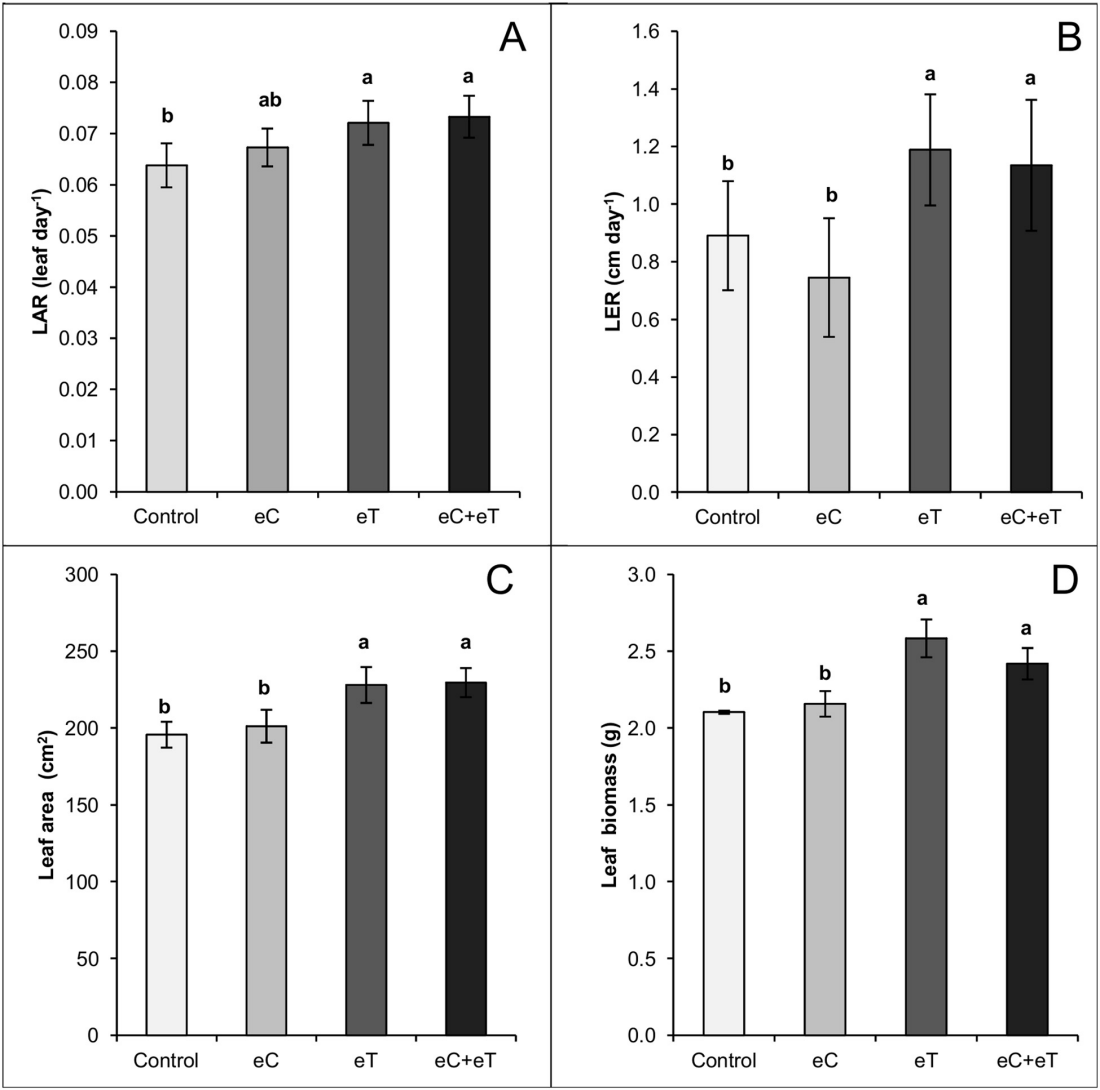
Leaf appearance rate (A), leaf elongation rate (B), leaf area (C), and leaf biomass (D) per tiller of *Panicum maximum* under regular CO_2_ and canopy temperature (Control), under elevated CO_2_ concentration of 600 ppm (eC), under elevated canopy temperature of +2°C (eT), and under both treatments (eC+eT). Bars show average values and lines at the top of the bars show the standard error. Different letters above bars indicate significant differences among datasets according to a Mann-Whitney test at p < 0.05.

The LAR was significantly higher (p < 0.05) in the eT and eC+eT treatments than the Control treatment (Fig 4A). The LER was also significantly higher (p < 0.05) in the eT and eC+eT treatments than in the control (Fig 4B). Therefore, leaves took more time to appear on tillers in the Control treatment and grew more slowly than in the eC treatment. This foliar dynamics explains the lower leaf area (Fig 4C) and leaf biomass (Fig 4D) in the Control and eC treatments than in the eT and eC+eT treatments. Leaf area was 17% higher in the eT and eC+eT treatments than the Control (Fig 4C). The leaf biomass was 22% and 15% higher in the eT and eC+eT treatments, respectively, than in the Control (Fig 4D). Therefore, in relation to the Control, the eC treatment applied alone did not significantly change LAR and LER (Fig 4A and 4B) and the foliar area and leaf biomass (Fig 4C and 4D).

**Fig 5:**
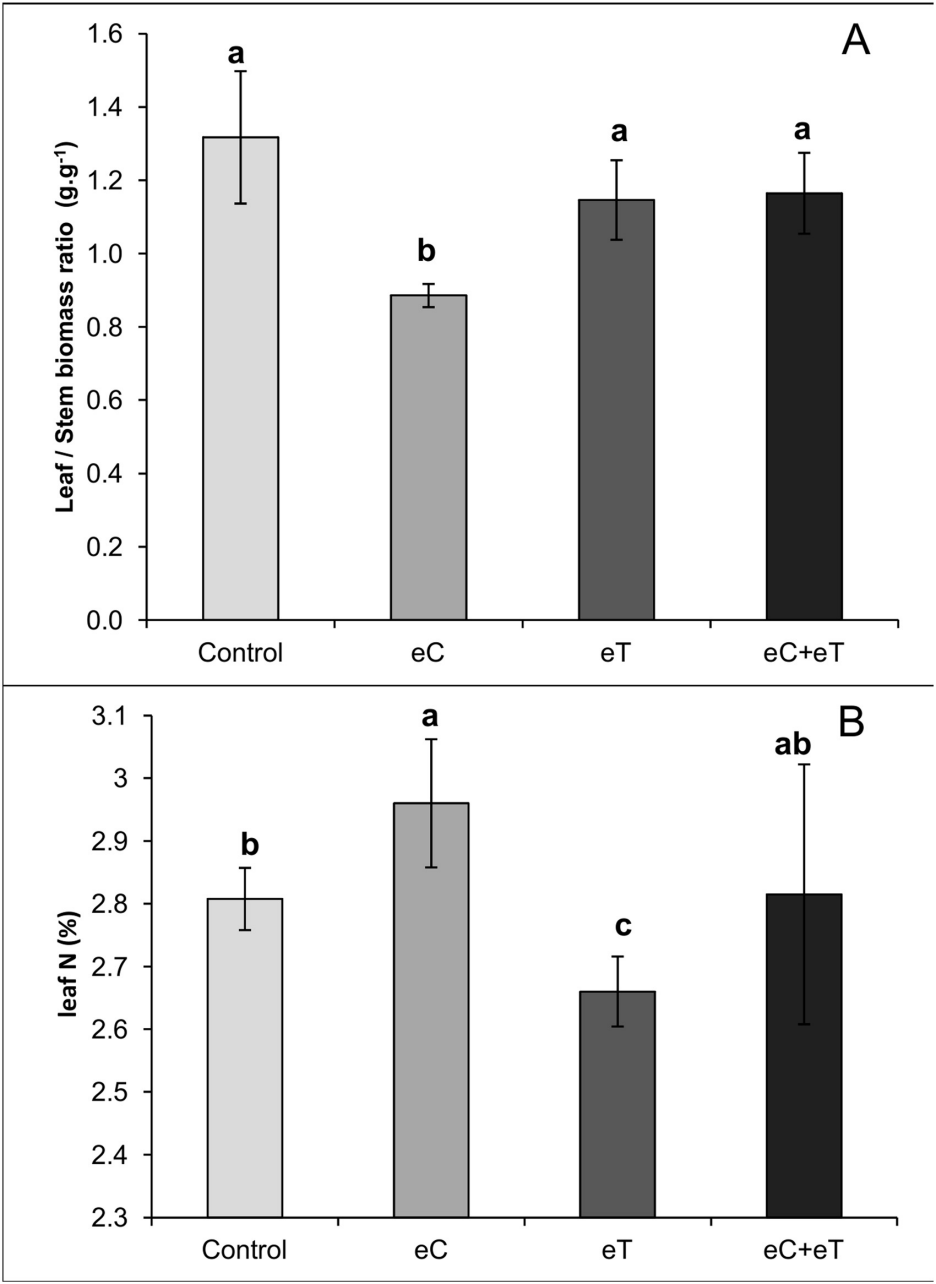
The leaf/stem biomass ratio (A) and leaf nitrogen content (B) of *Panicum maximum* under regular concentration of CO_2_ and canopy temperature (Control), under elevated CO_2_ concentration of 600 ppm (eC), under elevated canopy temperature of +2°C (eT), and under both treatments (eC+eT). Bars show average values and lines at the top of the bars the standard error. Different letters above the bars indicate significant differences among the datasets after the Mann-Whitney test at p < 0.1.

**Fig 6:**
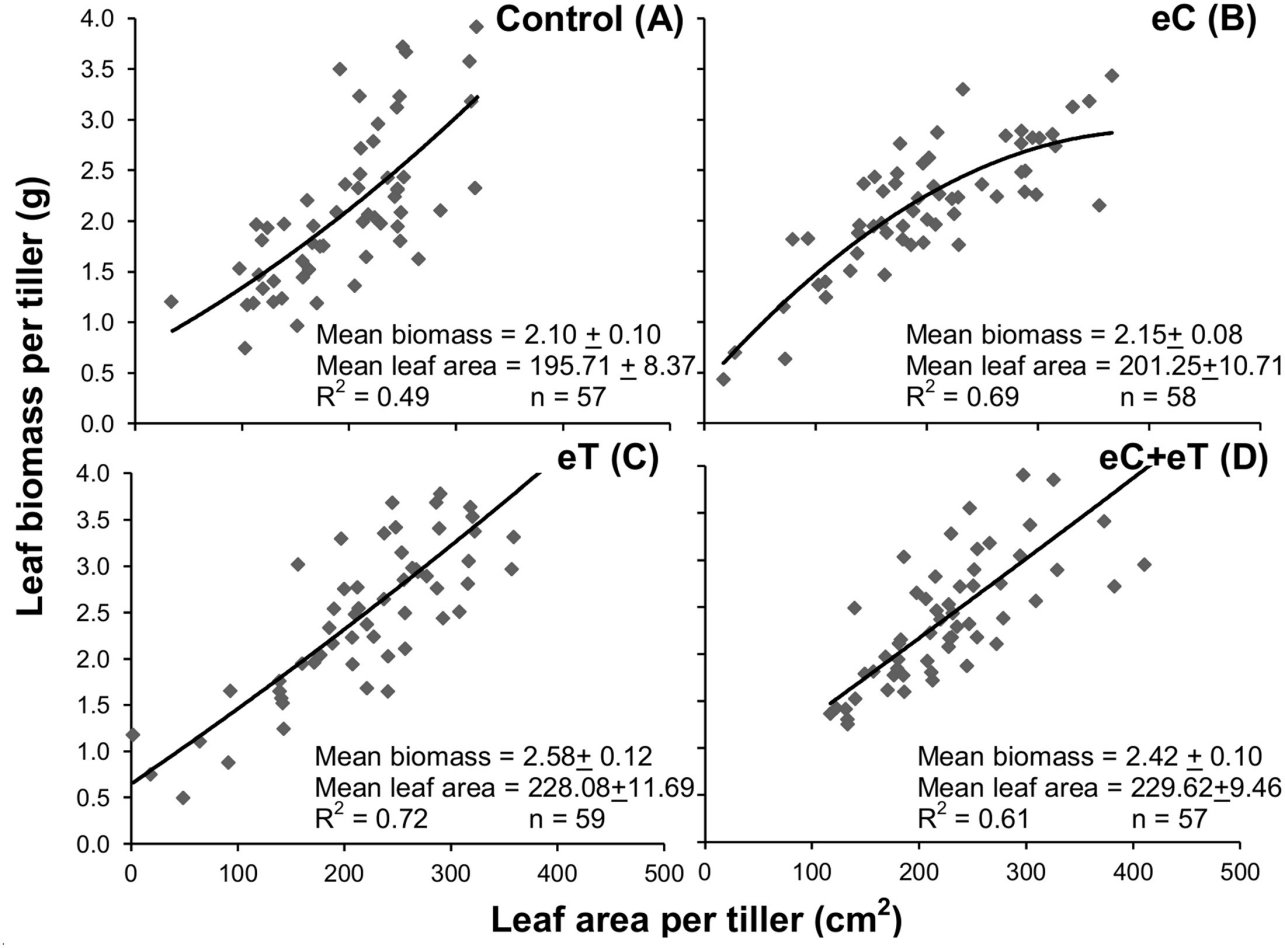
Average values of leaf biomass as a function of leaf area per tiller of *Panicum maximum* under regular atmospheric CO_2_ concentration and canopy temperature (Control, A), under elevated atmospheric CO_2_ concentration of 600 ppm (eC, B), under elevated canopy temperature of +2°C (eT, C), and under both treatments (eC+eT, D).

Fig 5 shows that the eC treatment had the lowest leaf/stem ratio on a mass basis (Fig 5A). Therefore, it is clear that the eC treatment favored stems. In addition, the highest and lowest leaf nitrogen content were in the eC and eT treatments, respectively (Fig 5B). This contrasting leaf nitrogen content could be a result of the dilution and concentration in foliage caused by the highest and lowest LER under the eT and eC treatments, respectively (Fig 4B). In the Control treatment, there was a nonlinear increase in leaf biomass as a function of leaf area (Fig 6A). However, in the eC treatment the leaf biomass-area relationship had a rectangular hyperbolic response with a higher degree of correlation (Fig 6B). Alternatively, leaf biomass and area had a linear relationship in the eT treatment (Fig 6C and 6D). This indicates that there were important differences in leaf blade formation among treatments.

**Fig 7:**
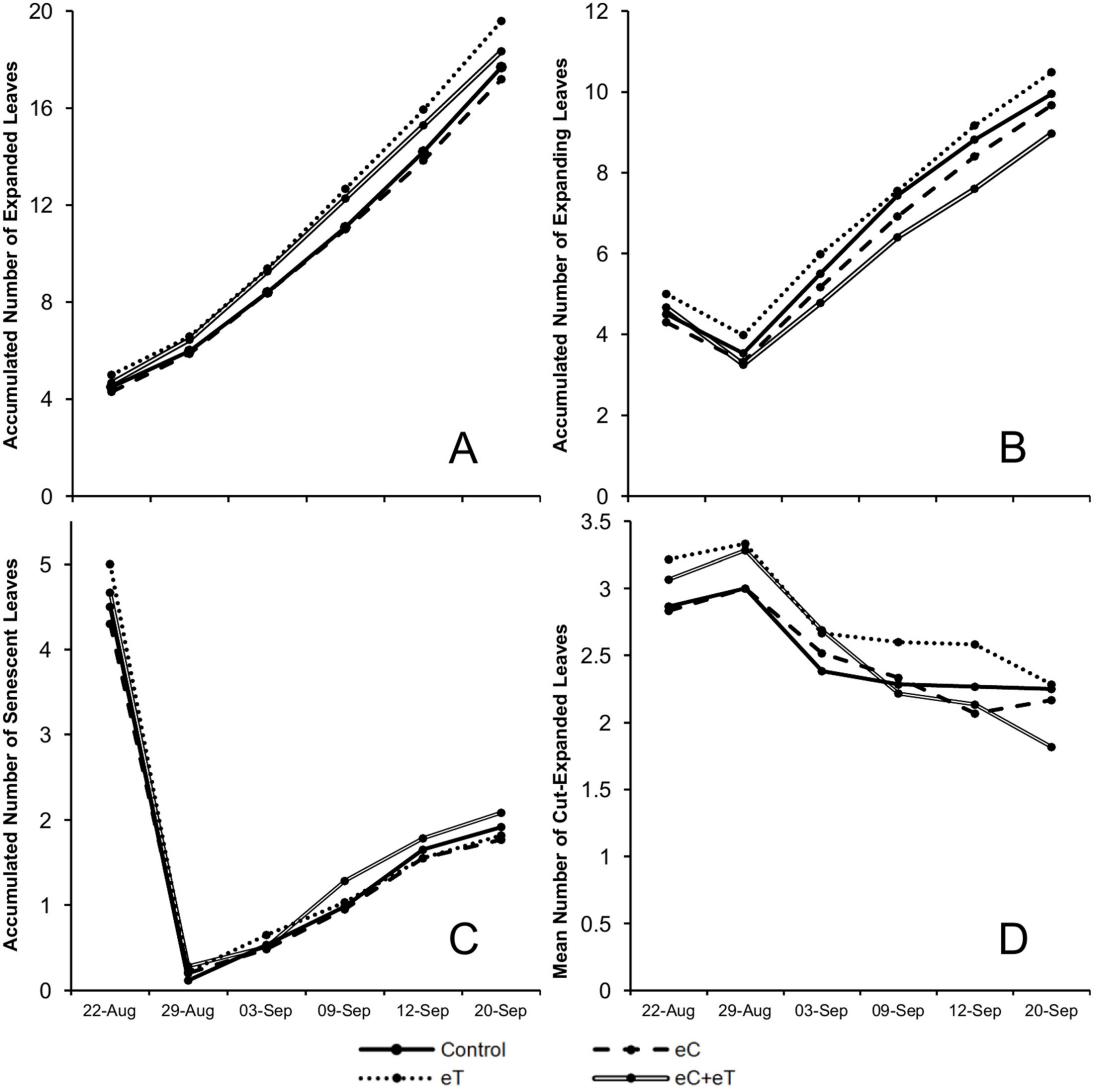
Accumulated number of expanded (A), expanding (B), and senescent (C) leaves; and the mean number of cut-expanded leaves (D) per tiller of *Panicum maximum* growing under different atmospheric conditions. A—Regular concentration of CO_2_ and canopy temperature (Control). B—Elevated CO_2_ concentration of 600 ppm (eC). C—Elevated canopy temperature of +2°C (eT). D—Combination of treatments (eC+eT). The days of measurement were August 22 and 29, and September 3, 9, 12, and 20, 2013.

At the end of the experiment, the eT treatment had the most expanded and expanding leaves (Fig 7A and 7B, respectively), and the eC treatment had the most senescent leaves. In addition, the eC treatment had the fewest cut-expanded leaves at the end of the experiment. Therefore, the variables that represented foliage gain (Fig 7A and 7B) were highest in the eT treatment. Besides, high foliage loss, represented by the many senescing leaves and few cut-expanded leaves, took place in the eC treatment (Fig 7C and 7D). However, it is notable that the eC treatment had the fewest expanding instead of expanded leaves from August 29 to September 20 (Fig 7A and 7B).

## Discussion

In C4 species, net photosynthesis has a peak value between 30°C–40°C and is only half of the maximum at 20°C [29]. In fact, the optimum temperature values for *Panicum coloratum* were 36.1°C and 38.1°C under high (35/30°C day/night) or moderate (25°C/20°C day/night) temperature regimes [16]. Therefore, in this experiment *Panicum maximum* grew under suboptimal temperatures for photosynthesis during the day throughout the experiment, since the maximum temperature did not reach 35°C even at midday. Thus, an increase of 1°C–2°C above the regular temperature during winter (Fig 2) probably led to a significant difference in the carbon net assimilation after 30 days in *Panicum maximum*. Further, the increase in leaf temperature towards the optimum for photosynthetic activity promoted foliage production (Figs 3A, 3B, 3C, 4, 7A and 7B). Besides, eT did not favor leaf senescence or limit the survival of cut-expanded leaves. Consequently, high leaf area and leaf biomass was observed when eT was applied alone or in combination with eC. Indeed, since the eT treatment had the most expanded and expanding leaves, it had the highest number of green leaves per tiller.

The benefits of elevated CO_2_ to C4 crop species are controversial. C4 crops benefit from elevated CO_2_ only under drought that causes low stomatal conductance and an increased concentration of intercellular CO_2_ and water use efficiency [30]. However, the supply of C4 acids may exceed the carboxylation capacity, resulting in the leaking of CO_2_ from the bundle sheath under a high atmospheric CO_2_ concentration [31]. In this sense, the downregulation of the molecular components of C4 carbon assimilation may occur under a high atmospheric CO_2_ concentration. Actually, the carboxylation efficiency and CO_2_ saturation rate of photosynthesis were lower in the C4 crop *Sorghum bicolor* grown free of water stress at elevated CO_2_ (700 ppm, growth chamber), accompanied by a 49% reduction in the phosphoenolpyruvate carboxylase content of leaves [32]. In addition, the carboxylation efficiency and the activities of C4 enzymes in *Zea mays* were reduced in a growth chamber with CO_2_ enrichment at 750 ppm [33].

An increase in leaf area and leaf biomass allocation are not expected to be concomitant with the downregulation of photosynthetic enzymes under high CO_2_ concentrations. Indeed, the leaf area and LAR did not change in C4 *Zea mays* under 750 ppm in five different day/night temperature regimes in a growth chamber without water stress, ranging from 19/13 to 38.5/32.5°C [33]. In our experiment using Trop-T-FACE, plants grown under elevated CO_2_ also were not significantly different from the control in LAR, LER, leaf area, and leaf biomass (Fig 4). Elevated CO_2_ could be beneficial at suboptimal temperatures for net photosynthesis by raising the leaf temperature through low stomatal conductance and transpiration [30]. Even so, if leaf temperature rose under eC, it was not beneficial in terms of leaf area or biomass production (Fig 4C and 4D).

On the other hand, the C4 *C*. *ciliaris* significantly increased biomass production and had a three-fold increase in the leaf area index after 120 days under 600 ppm of CO_2_ in an open top chamber [12]. In another experiment, three cultivars of *C*. *ciliaris* (Biloela, Aridus, and West Australian) were exposed to 370 and 550 ppm of CO_2_ for 50 days in a growth chamber [13]. The West Australian cultivar had significantly more forage mass and a higher shoot/root ratio in 550 ppm. In addition, there was an increase in the total dry mass under different N supplies in *Panicum coloratum* and, especially, *C*. *ciliaris* grown under 850 ppm CO_2_ in a growth chamber [31].

Biomass gain results in studies with FACE systems are lower than those in CO_2_ chamber studies [14] [34]. While FACE systems allow plant exposure to elevated CO_2_ under field conditions, growth chambers (phytotrons) and open top chambers (OTC) eliminate natural factors, such as wind and a broader daily range of leaf temperature. In addition, phytotrons and OTC limit the volume of soil for root growth [14], altering an important source-sink relationship in carbon metabolism [34]. Besides, OTC studies have had a mean CO_2_ concentration of 700 ppm, while studies with FACE use 550–600 ppm [35].

There is evidence that the eC treatment inhibited foliage development even though it was not different from the Control in LAR, LER, leaf area, or leaf biomass. The eC treatment yielded a significant decrease in the leaf/stem biomass ratio (Fig 5A). Leaf biomass was exponentially related to leaf area in the Control; however, a rectangular hyperbolic response was observed in the eC treatment limiting the increment of leaf dry biomass to below 3.0 g per tiller (Fig 6A and 6B). Indeed, the fewest green (Fig 3A) and expanded (Fig 3A) leaves during the experiment and the fewest accumulated expanded leaves (Fig 7A) were found in the eC treatment.

Elevated CO_2_ could affect leaf quality beyond leaf population dynamics and biomass partitioning in winter. On one hand, the impairments in leaf growth in eC resulted in a high leaf N concentration (Fig 5B), which could increase the nutritional value of such foliage. On the other hand, the eC treatment altered the biomass-area relationship in leaves, limiting the amount of biomass for expanded leaves. This means that large leaves in the eC treatment tended to have a thinner mesophyll cross-section. The leaf morphological trait most associated with animal preference is leaf width, a finding attributed to the wider mesophyll cross section [15], [36]. Instead, the limitation in the biomass gain of large leaves imposed by the eC treatment did not appear in the eC+eT treatment (Fig 6C and 6D).

Higher temperatures could compensate for the inhibitory effect of elevated CO_2_ by increasing the number of expanded leaves (Fig 3B). However, the synergistic inhibitory effect of the two resulted in the eC+eT treatment having the fewest expanding leaves during the experiment (Fig 3C). In fact, the mean number of cut-expanded leaves decreased in all treatments owing to the senescence with no possibility of replacement of such leaves shortly after pruning (August 29). However, the decrease in the population of cut-expanded leaves was higher in the eC+eT treatment (Fig 7D). This indicates that future increases in temperature and CO_2_ will speed up the senescing of leaves in *P*. *maximum* growing free of water and nutrient shortage in winter. This was confirmed by the highest number of accumulated senescent leaves in the eC+eT treatment between September 9–20 (Fig 7C). Indeed, the accumulated number of young leaves, such as expanding leaves, was lowest in the eC+eT treatment from September 3 through the end of the experiment (Fig 3B). Therefore, the influence of the eC+eT treatment on early ligule formation resulted in a high accumulated number of expanded and senescent leaves (Fig 7A and 7C, respectively).

In summary, the most green, expanding, and expanded leaves and the highest LAR and LER occurred in the eT treatment. In addition, heated plots had significantly higher leaf area and leaf biomass. The increase in LER and LAR without a change in the number of senescent leaves could explain how tillers were able to produce large foliage area and leaf biomass under elevated temperatures. Contrastingly, the eC treatment had the lowest LER and the fewest expanded and green leaves, with no substantial differences from the Control. The possible inhibitory effect of elevated CO_2_ on foliage development in winter was noticeable when comparing the eT and eC+eT treatments, since there were fewer green, expanded, and expanding leaves in the eC+eT treatment. The corresponding stimulatory and inhibitory effects on foliage of the eT and eC treatments resulted, respectively, in the dilution and concentration of foliar nitrogen. Despite no difference in leaf area and leaf biomass between the Control and eC treatments, the inhibitory effects of elevated CO_2_ on foliage could be confirmed by the eC treatment having the lowest leaf/stem biomass ratio. The change in leaf biomass-area relationships from linear or exponential growth to rectangular hyperbolic growth in the eC treatment also showed the inhibitory effect of elevated CO_2_ on foliage. Therefore, the relationship between eC and eT was not antagonistic, but at least once had a synergistic effect in accelerating leaf maturation.

## Conclusions

Several levels of organization related to leaves will significantly affect *P*. *maximum* under future climatic change, at least in winter. Elevated temperatures and CO_2_, or their combination, will alter leaf morphology, development, phenology, and autotrophic/heterotrophic biomass partitioning. Leaf area-mass relationships will become linear as temperatures rise or develop a clear hyperbolic relationship with a limit below 3.0 g of dry matter per tiller under elevated CO_2_. With sufficient water and nutrients, elevated winter temperatures will result in higher leaf expansion and appearance rates. More green leaves and a higher leaf area and biomass per tiller will occur with rising temperatures if *P*. *maximum* grows free of drought and nutritional stresses in winter. Contrastingly, when combined with elevated temperatures, elevated CO_2_ will inhibit foliage formation by favoring biomass partitioning to stems or by speeding up leaf maturation. Despite the CO_2_-temperature synergism, elevated temperatures can partially offset the inhibitory effects of CO_2_ on leaf development and biomass partitioning, leading to a higher foliage area and biomass than in current atmospheric conditions. The significant changes in the mass-area relationship and nitrogen concentration in leaves under elevated CO_2_ will very likely alter, respectively, the leaf palatability and leaf functioning of *P*. *maximum*.
